# Clinicopathological characteristics of gastrointestinal schwannomas: A retrospective analysis of 78 cases

**DOI:** 10.3389/fonc.2022.1003895

**Published:** 2022-12-13

**Authors:** Hailing Peng, Liu Han, Yuyong Tan, Yi Chu, Liang Lv, Deliang Liu, Hongyi Zhu

**Affiliations:** ^1^ Department of Gastroenterology, The Second Xiangya Hospital of Central South University, Changsha, Hunan, China; ^2^ Research Center of Digestive Disease, Central South University, Changsha, Hunan, China

**Keywords:** gastrointestinal schwannomas, submucosal tumors, endoscopic therapy, surgical treatment, prognosis

## Abstract

**Introduction:**

Schwannomas are tumors arising from Schwan cells of the neural sheath, which rarely occur in the gastrointestinal tract. The aim of the present study was to analyze the clinicopathological features and treatment outcomes of gastrointestinal schwannomas (GISs).

**Methods:**

Patients who were diagnosed with GISs in our hospital from January 2010 to December 2021 were selected. Data about demographic characteristics, clinical symptoms, treatment methods and outcomes, pathological results, and follow-up results were retrospectively collected and analyzed.

**Results:**

A total of 78 patients with 79 GISs were included, the female-to-male ratio was 55:23, and the average age was 52.12 ± 12.26 years. One-third (26/78) of the patients were asymptomatic. A total of 79 GISs were removed, and the average size was 3.63 ± 2.03 cm (range, 0.3–10 cm). As for tumor location, 54 GISs were located in the stomach, 14 in the esophagus, 2 in the duodenum, 6 in the colorectum (4 in the colon and 2 in the rectum), and the other 3 in the small intestine. A total of 23 and 55 patients underwent endoscopic and surgical resections, respectively. Compared with surgical resection, endoscopic resection is associated with a smaller diameter, lower cost, and shorter hospital stay. Pathological results revealed that S100 was positive in all the GISs. No recurrence was noticed during a median follow-up of 45 months (range, 6–148 months).

**Conclusion:**

GISs are rare gastrointestinal tumors with favorable prognoses, which are most commonly seen in the stomach and diagnosed by pathological findings with immunohistochemical staining. Surgical resection remains the standard method for removing GISs, while endoscopic resection may serve as an alternative method for selected patients with GISs and may be attempted in GISs with a diameter of <3 cm and no signs of malignancy.

## Introduction

With the wide application of esophagogastroduodenoscopy, colonoscopy, and endoscopic ultrasonography (EUS), the detection rate of gastrointestinal submucosal tumors (SMTs) has increased obviously (1). Gastrointestinal SMTs, also called mesenchymal tumors, comprise 0.1% to 3% of all gastrointestinal tumors, and they consist of a spectrum of spindle cell tumors, mainly including gastrointestinal stromal tumor (GIST), leiomyoma or leiomyosarcoma, and schwannoma (2). Schwannomas arise from the Schwann cells in nerve sheaths, which grow slowly and can occur in any part of the body but are rarely seen in the gastrointestinal tract (3, 4). GIS was first reported by Daimaru in 1988 (5), and it is being diagnosed more frequently with recent advances in diagnostic technology and immunohistochemistry (IHC). Although computed tomography (CT) and EUS may provide useful information in diagnosis and differentiating GISs from other SMTs, such as GIST (6–8), confirmed diagnosis relies on histological and IHC results.

Removal of the tumor is recommended for symptomatic and large gastrointestinal SMTs (≥2 cm), and periodic surveillance is suggested for asymptomatic and small ones (<2 cm) (1). However, patients usually feel stressed, and some patients cannot adhere to the surveillance strategy. In fact, the surveillance itself is associated with repeated endoscopic procedures and a risk of a delayed diagnosis of malignancy. Moreover, GISs are regarded as potential malignant tumors, as malignant GISs have been reported (9–15), although the majority of GISs are benign. Therefore, most of the patients with gastrointestinal SMTs choose to remove them upon detection. Surgical resection is the standard method for the treatment of GISs; endoscopic resection has been reported as an alternative strategy for selected patients (usually for size <3 cm) (16, 17).

Currently, most of the studies focused on gastric schwannomas, and few studies reported the GISs in the whole gastrointestinal tract (18, 19), and the sample size was relatively small. In the present study, we retrospectively collected and analyzed the clinical data of GISs diagnosed in our hospital to present the clinicopathological characteristics and treatment outcomes of this rare disease.

## Materials and methods

### Patients

This is a retrospective study conducted in a tertiary hospital in China and was approved by the ethics committee of the Second Xiangya Hospital of Central South University. All the patients or legal guardians provided signed informed consent before the procedure was performed. The inclusion criteria of the study were as follows: 1) GIS confirmed by postoperative histological and IHC results, 2) patients who underwent endoscopic or surgical resection at our hospital, and 3) complete medical records. Exclusion criteria were as follows: 1) patients who were diagnosed with GIS preoperatively but did not receive endoscopic or surgical resection; 2) patients who underwent endoscopic or surgical resection at other hospitals but sent the specimen to our hospital for a confirmed diagnosis. Their demographics (age and gender), tumor-related parameters (location, size, IHC results, etc.), treatment methods, complications, hospital stay, and follow-up data were retrospectively collected and recorded.

Telephone calls and outpatient interviews were used for follow-up. CT or endoscopy was performed every 6 months during the first year and annually thereafter.

### Statistical analysis

SPSS 25.0 software (SPSS, version 25.0, IBM Corp., Armonk, NY, USA) was applied for data analysis. Continuous variables were presented as mean ± standard deviation and analyzed using Student’s t-test. Qualitative data were presented as frequencies and calculated using the chi-square test or Fisher’s exact test. A two-tailed p-value of <00.05 was considered statistically significant in all cases.

## Results

### General clinical characteristics

From January 2010 to December 2021, a total of 88 patients were diagnosed with GIS in the pathological database of our hospital, among whom four patients confirmed the diagnosis of GIS by biopsy but refused to have the lesions removed, and six patients underwent surgical resection at other hospitals and sent the specimen to our hospital for a confirmed diagnosis. Therefore, a total of 78 patients were included. Within the same period, a total of 2,104 patients with gastrointestinal SMTs (982 in the stomach, 631 in the esophagus, 136 in the duodenum, 132 in the small intestine, and 223 in the colorectum) were treated in our hospital; therefore, GISs account for 3.71% of all the gastrointestinal SMTs.

**Figure 1:**
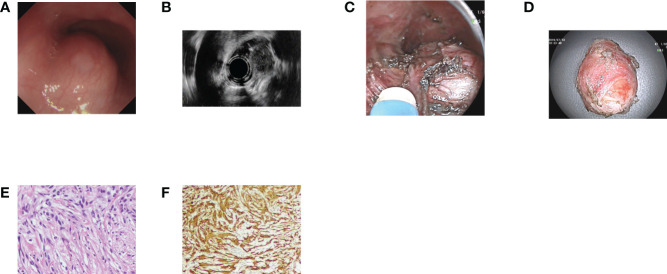
Case illustration of an esophageal schwannoma. **(A)** A submucosal tumor was seen in the esophagus. **(B)** Endoscopic ultrasonography revealed that the tumor originated from the muscularis propria layer with heterogeneous echo. **(C)** The tumor was removed by submucosal tunneling endoscopic resection, and we could see the tumor in the submucosal tunnel. **(D)** The resected tumor. **(E)** Histological results revealed spindle cell tumors. **(F)** Immunohistochemical staining of S100 was positive, consisting of schwannoma.

**Figure 2:**
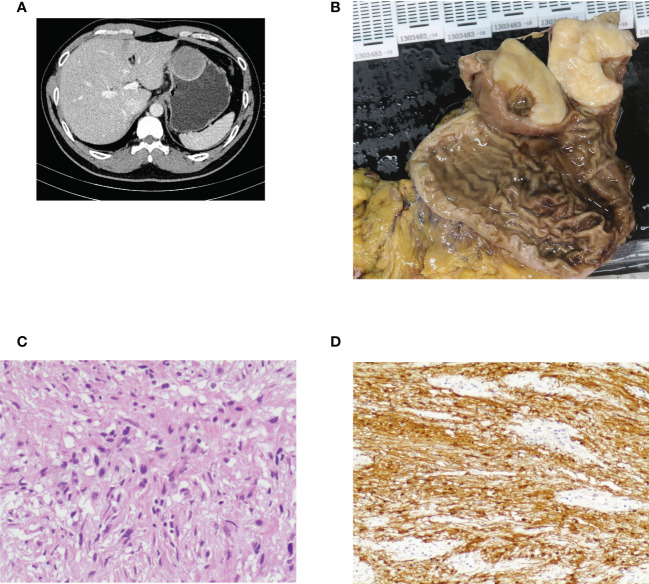
Case illustration of gastric schwannoma. **(A)** Computed tomography showed a protruding lesion in the stomach. **(B)** The tumor was removed by surgical resection, and this was the resected tumor. **(C)** Histological results revealed spindle cell tumors. **(D)** Immunohistochemical staining of S100 was positive, consisting of schwannoma.

Among the 78 patients, 55 were female and 23 were male. The average age was 52.12 ± 12.26 years (range, 20–80 years). A total of 79 GISs were removed, and the average size was 3.63 ± 2.03 cm (range, 0.3–10 cm). As for tumor location, 14 were in the esophagus (**Figure 1**), 54 in the stomach (**Figure 2**), 2 in the duodenum, 6 in the colorectum (4 in the colon and 2 in the rectum), and the other 3 in the small intestine (**Table 1**). Of the 54 GISs in the stomach, 39 were located in the gastric body, 6 in the gastric fundus, 3 in the gastric angle, and 6 in the antrum. For the 14 GISs in the esophagus, 6 were in the upper esophagus, 5 were in the middle esophagus, and 3 were in the lower esophagus.

**Table 1:** Clinicopathological characteristics of the 78 patients with GISs.

Variables	
Gender	
Female	70.5% (55/78)
male	29.5% (23/78)
Average age, years (range)	52.12 ± 12.26
<40	12.7% (10/78)
40–60	60.8% (48/78)
>60	25.3% (20/78)
Clinical presentation	
Asymptomatic	33.3% (26/78)
Abdominal pain	37.3% (29/78)
Abdominal discomfort	6.4% (5/78)
Abdominal distension	5.1% (4/78)
Gastrointestinal bleeding	2.6% (2/78)
Other symptoms	15.4% (12/78)
Major comorbidities	
No comorbidities	38.5% (30/78)
Hypertension	20.5% (16/78)
T2DM	6.4% (5/78)
Gastrointestinal polyps	12.8% (10/78)
Gastrointestinal cancers	6.4% (5/78)
Tumor size, cm (range)	3.63 ± 2.03
Tumor location	
Esophagus	17.7% (14/79)
Stomach	68.4% (54/79)
Duodenum	2.5% (2/79)
Colon	5.1% (4/79)
Rectum	2.5% (2/79)
Small intestine	3.8% (3/79)
Originate layer	
Submucosal layer	7.6% (6/79)
Muscularis propria layer	92.4% (73/79)
Growth pattern	
Intraluminal	62.0% (49/79)
Extraluminal	22.8% (18/79)
Mixed	15.2% (12/79)
Treatment methods	
Endoscopic resection	29.5% (23/78)
Surgical resection	70.5% (55/78)
Histological results	
Benign	97.5% (77/79)
Malignant	2.5% (2/79)
Median follow-up, months (range)	45 (6–148)

GISs, gastrointestinal schwannomas; T2DM, type 2 diabetes mellitus.

A total of 48 patients had comorbidities, 5 of them had coexisting tumors (namely, 2 colon cancer, 1 esophageal cancer, 1 pancreatic cancer, and 1 GIST), and 10 had coexisting gastrointestinal polyps. A total of 52 patients had symptoms, and the most common symptoms were abdominal pain (29/78). A total of 26 patients were asymptomatic, and their GISs were found accidentally by endoscopy or CT examination. A total of 70 and 5 patients underwent esophagogastroduodenoscopy and colonoscopy preoperatively, respectively, and only 2 patients with gastric schwannomas showed ulceration in the covering mucosa, while the others showed intact mucosa. A total of 38 patients underwent EUS examination preoperatively, and echo analysis revealed that 36 of them were low echoes (14 were homogeneous and 22 were heterogeneous), and 2 were medium-high echoes. A total of 62 patients received CT examination preoperatively, among whom 28 showed homogeneous tumors; 16 patients showed heterogeneous, slow, and progressive enhancement; and 3 patients showed strong enhancement. However, others did not describe such information or received a non-contrast CT examination. Only one patient showed necrosis on CT scans. As for the growth pattern, 49 of the 79 GISs were intraluminal growth, 18 were extraluminal, and the remaining 12 were mixed.

### Pathological and immunohistochemistry results

All the patients confirmed the diagnosis of GISs postoperatively *via* pathological and IHC staining. A total of 77 GISs from 76 patients were diagnosed with benign GISs, and only 2 patients were considered malignant. S100 staining was positive in all the patients, while SOX-10, Vimentin, Dog-1, CD34, SMA, and CD117 were positive in 97.2% (35/36), 100% (32/32), 0% (0/68), 24.2% (18/74), 17.9% (14/78), and 6.4% (5/78), respectively. Ki-67 was performed in 70 patients and was positive in 63 patients, the index was 1%–10% in most of the patients (61/63), and only the 2 patients diagnosed with malignant GIS had a Ki-67 index of >10%.

### Treatment outcome

A total of 23 and 55 patients underwent endoscopic resection and surgical resection, respectively. A total of 79 GISs were removed, and 74 were in the muscularis propria layer and 5 in the submucosal layer. Among the 23 patients who received endoscopic resection, 4 underwent endoscopic mucosal resection, 6 endoscopic submucosal dissections, 4 endoscopic submucosal excavation, 4 endoscopic full-thickness resections, and 5 submucosal tunneling endoscopic resection. Of the 55 patients who received surgical resection, 20, 24, 7, 2, 1, and 1 received laparoscopic, open, robot-assisted, thoracoscopic, laparoscopic, and endoscopic cooperative surgery and transanal endoscopic microsurgery, respectively. Compared with patients who underwent surgical resection, patients who underwent endoscopic resection had a small tumor size (1.87 ± 1.36 vs. 4.38 ± 1.82 cm) but a lower cost (22,905.12 ± 12,711.75 vs. 62,336.29 ± 31,044.19 yuan) and shorter postoperative stay (7.48 ± 5.29 vs. 10.25 ± 4.46 days) (**Table 2**). A total of 13 patients encountered complications, namely, 4 pulmonary infections, 3 peritonitis, 2 gastrointestinal bleeding, 1 wound infection, 1 pulmonary embolism, 1 anastomotic stricture, and 1 patient with hydropneumothorax and esophagomediastinal fistula; no patient died of these complications. During a median follow-up of 45 months (range, 6–148 months), no recurrence was noticed. Two patients died during follow-up, and GIS was not the cause of death (one died from pancreatic cancer and the other from coronary heart disease).

**Table 2:** Comparison of endoscopic and surgical treatment of GISs.

Variables	Endoscopic resection	Surgical resection	*X* ^2^/t	p
	(n = 23)	(n = 55)	
Gender, male/female	Sep-14	14/41	1.459	0.227
Average age, years	56.30 ± 10.22	54.55 ± 10.85	−3.110	0.003
Tumor location			15.922	0.003
Esophagus	10	4		
Stomach	11	42		
Duodenum	0	2		
Colorectum	2	4		
Small intestine	0	3		
Tumor size, cm	1.87 ± 1.96	4.38 ± 1.82	−5.958	<0.001
Complications	13.0% (3/23)	18.2% (10/55)	0.308	0.579
Postoperative hospital stay, days	7.48 ± 5.29	10.25 ± 4.46	−2.371	0.02
Cost, yuan	22,905.12 ± 12,711.75	62,336.29 ± 31,044.19	−5.871	<0.001

GISs, gastrointestinal schwannomas.

## Discussion

In the present study, we summarized the clinical data of 78 cases of GISs and demonstrated that GISs are rare gastrointestinal tumors and most commonly seen in the stomach. Both endoscopic and surgical resections are acceptable for selected patients with favorable prognoses. As far as we know, this is the largest single-center report about GISs until now.

GISs are rare gastrointestinal mesenchymal tumors and account for about 2%–6% of all gastrointestinal mesenchymal tumors (3). GISs are most commonly located in the stomach (60%–80%), followed by the colon and rectum, and are even rare in the esophagus and small intestine (3, 19). In the present study, GISs account for 3.71% of gastrointestinal SMTs, and 67.9% (53/78) of them were located in the stomach, which was consistent with previous studies. However, GISs located in the esophagus (17.9%, 14/78) were more than those in the colorectum (7.7%, 6/78) in our study. Gastric schwannomas are more commonly located in the gastric body (56.5%–90.3% as reported) (6, 7, 17, 20–25), and 72.2% (39/54) were located in the gastric body in our study. GISs occur more frequently in women, with a female-to-male ratio of up to 2:1 or higher (3, 4, 19). The female-to-male ratio was 2.39:1 (55:23), which is in accordance with that found in the literature. GISs are more common among the elderly, especially for patients who are 40 to 60 years old. In the present study, the mean age was 52.12 years, 61.5% (48/78) of the patients were 40 to 60 years old, and only 12.8% (10/78) were younger than 40 years. Most of the GISs were asymptomatic and found incidentally; others may present non-specific symptoms such as abdominal discomfort or pain, gastrointestinal bleeding, and obstruction (3, 4, 19, 26). In the present study, 33.3% (26/78) were asymptomatic, and the most common symptom was abdominal pain.

Gastrointestinal endoscopy, EUS, and CT were useful for the detection of GISs and differentiating GISs from other gastrointestinal SMTs. Usually, the covering mucosa of GISs are smooth and intact; erosion or even ulceration was reported in few patients (0%–26.92%) (3, 4, 7, 8, 16, 22, 27). In the present study, 2.6% (2/78) had ulceration, while the other 76 had intact covering mucosa. Moreover, compared with GISTs, intralesional necrosis is rarely seen in GISs; in the present study, necrosis was only seen in one patient among the 62 patients who received a CT scan. Xu et al. (6) established a radiologic diagnostic scoring model to differentiate GISs and GISTs, which included four variables: transverse position (greater curvature), location (body or antrum), perilesional lymph nodes (present), and pattern of enhancement (homogeneous). EUS characteristics such as tumor location, gross morphology, layer of origin, echogenicity in comparison with the normal muscle layer, and presence of an internal echoic lesion were also useful to differentiate GISs from GISTs (7). GISs are more likely to present as low echo on EUS (87.7%–100%), originating from the muscularis propria layer (85.7%–100%) (7, 8, 16, 17, 21, 22). In the present study, 38 cases received preoperative EUS, and 36 (94.7%) of the GISs were presented as low-echo lesions; 93.7% (74/79) of the GISs originated from the muscularis propria layer, which is consistent with the literature.

Confirmed diagnosis of GISs depends on pathological findings with IHC results. S100 is a specific marker for GISs with a positive expression rate of 97.6%–100%. Other occasionally positive markers reported included Vimentin, CD34, and SOX10 (3, 4). Negative expression of other markers such as Dog-1, CD117, SMA, and desmin is useful for differentiating GISs from other mesenchymal tumors such as GIST and leiomyoma. However, CD117 (usually positive in GIST) and SMA (usually positive in leiomyoma) were positive in 6.4% (5/78) and 17.9% (14/78) of the patients, respectively, suggesting that a single IHC parameter could not differentiate GISs from GIST and leiomyoma, and a combination of several parameters is necessary. Other studies also reported a positive expression of CD117 and SMA in GISs (20, 22). No patient in the present study had c-KIT or PDGFRA mutation; therefore, we do not know the mutation status of c-KIT or PDGFRA in GISs. In the present study, the positive expression rate of S100 was 100%, and Dog-1 staining was negative in all the cases. We found that the expression of SOX-10 and Vimentin was positive in 97.2% (35/36) and 100% (32/32), respectively, which was rarely reported (22, 28–30), suggesting that these two markers may serve as important indicators for diagnosis of GISs (31). The Ki-67 index may help predict the malignancy of GISs; usually, Ki-67 > 10% is considered to be malignant (3). In the present study, Ki-67 was positive in 90% (63/70) of the patients, most of them were less than 10%, only 2 of them had a Ki-67 index > 10%, and they were diagnosed with malignant GISs histologically.

The treatment strategy of GISs is based on the size, location, and association with surrounding tissues; available treatment modalities include endoscopic resection and surgical resection (3). Currently, surgical resection remains the standard and most effective in treating GISs, and common surgical methods include simple tumor resection, partial gastric (intestinal and esophageal) resection, and subtotal/total gastrectomy (for gastric schwannomas). According to a literature review that included 319 cases of gastric schwannomas, endoscopic resection was only performed in 10% of the cases, while 44% received local surgery, and 46% received subtotal/total gastrectomy (4). Laparoscopic surgery is associated with superior to shorter operation time and postoperative hospital stay and less blood loss, compared with open surgery (21). In the present study, 20 received open surgery, 24 received laparoscopic surgery, and 7 received robot-assisted surgery. With the development of endoscopic techniques and equipment, some gastrointestinal SMTs can be successfully removed *via* endoscopic resection (32, 33). Several studies have demonstrated the feasibility, safety, and efficacy of endoscopic resection of gastric schwannomas (16, 17, 34–37). Zhai et al. (38) retrospectively analyzed 46 cases of gastric schwannomas (16 cases received endoscopic resection, and 30 received surgical resection) and found that patients in the endoscopic resection group had a shorter operative time and lower operation cost than those in the surgical resection group, while there was no significant difference in complete resection and adverse event rates and postoperative hospital stay, but the tumor size of endoscopic resection group was significantly lower than that in the surgical resection group (22.9 vs. 41.0 mm). In the present study, 23 patients received endoscopic resection, and the other 55 received surgical resection; the tumor size was larger in the surgical group; endoscopic resection was associated with a lower cost and shorter hospital stay; there was no significant difference in efficacy and complications. Therefore, endoscopic resection may serve as an alternative method for selected patients with GISs and may be attempted in GISs with a diameter of <3 cm and no signs of malignancy.

The present study has several limitations. First, this is a single-center, retrospective study. Second, only a portion of the patients received preoperative CT and/or EUS examination. Third, the IHC staining parameters were not exactly the same among patients; therefore, it is difficult to provide an exact positive proportion for some indicators. In conclusion, we found that GISs are rare gastrointestinal tumors with favorable prognoses and are most commonly seen in the stomach. Surgical resection is the standard method for removing GISs, while endoscopic resection may serve as an alternative method for selected patients with small GISs ≤3 cm.

## Data availability statement

The raw data supporting the conclusions of this article will be made available by the authors, without undue reservation.

## Ethics statement

The studies involving human participants were reviewed and approved by the ethics committee of the Second Xiangya Hospital of Central South University. The patients/participants provided their written informed consent to participate in this study.

## Author contributions

Research design: HP, LH, HZ. Administrative support and organization: DL. Data collection and analysis: HP, LH, YT, YC and LL. Manuscript drafting: HP, YT and HZ. Modification and final approval: All authors. All authors contributed to the article and approved the submitted version.
